# COVID-19: Measures to prevent hospital contagion. What do urologists need to know?

**DOI:** 10.1590/S1677-5538.IBJU.2020.S117

**Published:** 2020-07-27

**Authors:** Edgar Ivan Bravo Castro, Gerardo López Secchi, Cristobal Díaz Gómez, Javier Torres Gómez, Omar Clark, Ivan Azael Martinez Alonso, José Gadu Campos Salcedo

**Affiliations:** 1 Department of Urology Hospital Central Militar MéxicoDF México Department of Urology Hospital Central Militar, México DF, México; 2 Department of Urology Mutualista Asociación Española Montevideo Uruguay Department of Urology Mutualista Asociación Española, Montevideo Uruguay; 3 Department of Urology Hospital Francisco Soca Canelones Uruguay Department of Urology Hospital Francisco Soca, Canelones, Uruguay; 4 Hospital Central Militar Urology Section MexicoDF México Chief of the Urology, Urology Section, Hospital Central Militar, Mexico, DF, México

**Keywords:** Primary Prevention, Quarantine, COVID-19 diagnostic testing [Supplementary Concept]

## Abstract

A new outbreak of respiratory infection caused by the novel coronavirus in late December 2019 in China caused standards of medical care to change not only for related areas but for the entire healthcare system, and when the WHO declared COVID-19 a pandemic new strategies of patient care had to be defined initially to optimize resources to confront the pandemic and then to protect healthcare personnel. As urologists, we must be involved in these new standards, since without an effective vaccine the risk of contagion is high; thus, the purpose of this review is to have orientation on the measures urologists should take in their everyday clinical practice.

## INTRODUCTION

On December 31, 2019, 27 cases of pneumonia of unknown etiology were identified in the city of Wuhan, Hubei Province, in China. The characteristic manifestations seen in these patients included clinical symptoms of dry cough, dyspnea, fever, and bilateral pulmonary infiltrates in the images. All these cases were related with the Huanan wholesale seafood market in Wuhan, which sells fish and a wide variety of species of live animals, including poultry, bats, marmots, and snakes [1]. On January 7, 2020, the causal agent was isolated from nasopharyngeal swabs and was named SARS-CoV-2 by the World Health Organization (WHO), which causes the disease COVID-19. Although a majority of cases have resolved spontaneously, some have developed various fatal complications which include organ failure, septic shock, pulmonary edema, severe pneumonia, and acute respiratory distress syndrome (ARDS), which require management in an Intensive Care Unit (ICU) [1].

### Clinical characteristics of COVID-19

The SARS-CoV-2 virus is transmitted predominantly through aerosolization, particles from respiratory droplets or secretions; human-human transmission has been confirmed. As with any viral disease, maintaining a proper distance of at least 2 meters is one of the basic measures for prevention [2]. It is clear that the elderly and patients with chronic cardiopulmonary diseases are especially vulnerable, although numerous deaths have also been reported among patients age 50 years or less without comorbid conditions [3]. Various characteristics of the disease have been described as it advances through different stages. The disease COVID-19 is mediated by the virus bonding to “peak proteins” to coreceptors of the human Angiotensin-Converting Enzyme (ECA) concentrated in the lungs; however, they are also expressed in the brain, heart, kidneys, and gastrointestinal tract [4, 5].

### Optimizing resources

As hospitals start preparing for the possibility of high demand for care for cases of COVID-19, optimizing resources by cancelling elective surgeries will increase access to care [6]. Hard decisions have to be made on which surgeries should continue under present circumstances and elective procedures postponed until the pressure on the hospital system to provide care for patients with COVID-19 cases [6].

The choice of urgent or emergency surgery will depend on capacity and demand without overlooking diagnosis, and at the same time should be counteracted by the effects of delaying surgery and in particular genitourinary neoplasms and complicated lithiasic disease. Urologists can help by reducing demand for ventilators, personal protection equipment, and other critical hospital and human resources, minimizing surgeries without compromising patient outcomes whenever possible. The surgeries which should be prioritized to meet demand for care for COVID-19 and move forward are cases where evidence suggests that even short-term delays may affect the patient's survival. Also, alternatives are suggested for management of common urgent or emergency urological procedures which may prevent the use of ventilators, and we consider the use and impact of common urological treatments in patients during an infectious outbreak. Finally, although we do not incorporate the patient's age and fragility in these recommendations, the risk of postoperative COVID-19 infection and its potential impact on a patient's postoperative evolution should also be considered [7].

Shared decision making should be encouraged. To the extent possible, patients' holistic needs, like managing anxiety, should be considered when discussing decisions on postponements in treatment. To the extent possible, patients should be informed that decisions regarding elective cancer surgery are based on a consensus, are based on emerging data, and are based both on wanting to give them the best opportunities to achieve good outcomes in their cancer and minimize their risk of harm from COVID-19 [8].

### General aspects

Even if there is insufficient scientific evidence on managing care for patients with suspected or confirmed COVID-19 infection who require urological surgery, there are general aspects which make up good clinical practice in prevention of events which help minimize the risk of contagion in the different stages of the procedure [9].

The high level of diffusion of the pathogen and its virulence, which has pushed healthcare systems in different countries to the point of saturation, makes it essential to know and take the relevant steps correctly to prevent contagion to healthcare providers and prevent them from becoming potential transmitters of the disease, as is seen statistically in around 10-15% of cases.

The concerns and anxiety of healthcare professionals are constantly in these cases, which should be heard, protected, prepared, supported, and cared for [10]. This makes it necessary to oblige everyone circulating in a medical care institution to use a properly fitted facemask.

### Improving protection for the healthcare team

A healthy and effective HCP team is crucial to successfully prevent the ongoing epidemic from expanding further. The large number of infections underscores the need and urgency of protecting the COVID-19 healthcare team. It is praiseworthy that during the pandemic the Chinese government has placed high importance on protecting the health of healthcare personnel and has taken a series of immediate measures [11], like better orientation on proper use of personal protection equipment (PPE), better logistics and medical supplies, and better disinfection in hotels where healthcare personnel are housed. Also, there is now an emergency tracking system to monitor all healthcare personnel exposed, which contributes to rapid detection, effective classification and isolation of infected patients. A special group of medical experts are doing everything possible to diagnose and treat medical personnel with suspected and confirmed infection. Also, a special health and life insurance fund was created for all healthcare professionals working on the front line at the national and provincial levels [12]. All these factors help guarantee the confidence and efficiency of healthcare professionals, but there is still much to do to protect their long-term occupational health even more.

### Options for elective surgery

Preadmission and hospitalization of urology patients programmed for elective surgery during the COVID-19 pandemic should consider two important practical aspects, such as the need to reduce the home-to-hospital traffic flow and limited (null) access to all diagnostic tests related to preadmission. Also, it should be important to guarantee that patients coming from home do not constitute a source of contagion for hospitalized patients. Preoperative tests should be performed in a single hospital visit whenever possible, after telephone classification of COVID-19 symptoms and using preferential and well-defined hospital routes. Routine blood and instrumental tests to define the risk of anesthesiology should be performed in all patients programmed for elective surgery [13].

Specifically analyzing the ideal pathway, which is desirable in COVID-19-free hospitals in the pre-admission phase, it would be advisable to take nasopharyngeal swabs in all patients to rule out the presence of coronavirus 2 in severe acute respiratory syndrome (SARS) -CoV-2) as outpatients [14].

Based on the indications of the Centers for Disease Control and Prevention [14], it is advisable to guarantee a unique access point to facilitate detection procedures.

Excluding the risk of suspicious symptoms and signs (body temperature) of COVID-19, hospitalized patients should be asked to use a surgical mask and to observe the rules of hygiene recommended for the general population. A valid aid is to reduce the number of beds per room and/or guarantee the minimum safe distance between patients.

The recommendation to use individual protection systems is mandatory both for patients and for healthcare workers.

Bearing in mind that the majority of non-deferrable surgical procedures are performed to treat malignant genitourinary tumors [15], staging is of vital importance. In this context, although the recommendations of international guidelines should be respected, tests which are not essential for surgical planning and staging should be postponed.

### Approach in the emergency ward

**Figure 1:**
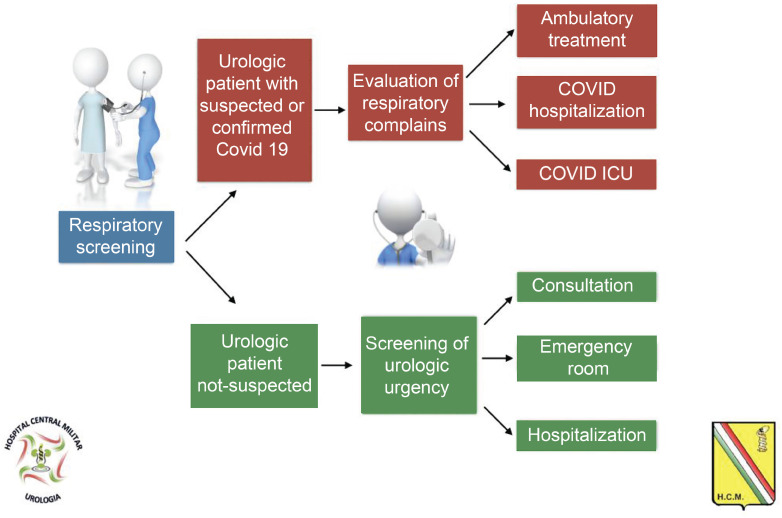
Approach to patients with suspected urologic emergency or with confirmed diagnosis of COVID-19 at the department of Urgency of the Hospital Central Militar.

At Hospital Central Militar in Mexico, from the onset of the pandemic, the facility was set up to receive patients with COVID-19 without failing to attend emergencies involving other disorders, including urological emergencies; to achieve that, care was set up as follows (Figure-1).

If surgical procedures are inevitable, it is recommended that all procedures be performed by experienced urologists.

**Figure 2 A and B:**
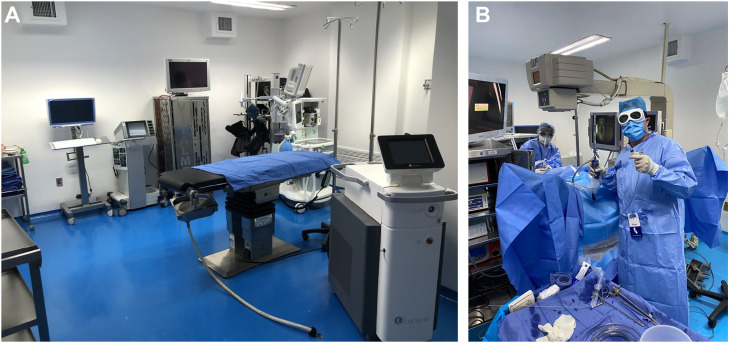
Intraoperative Management Hospital Central Militar, México.

**Figure 3:**
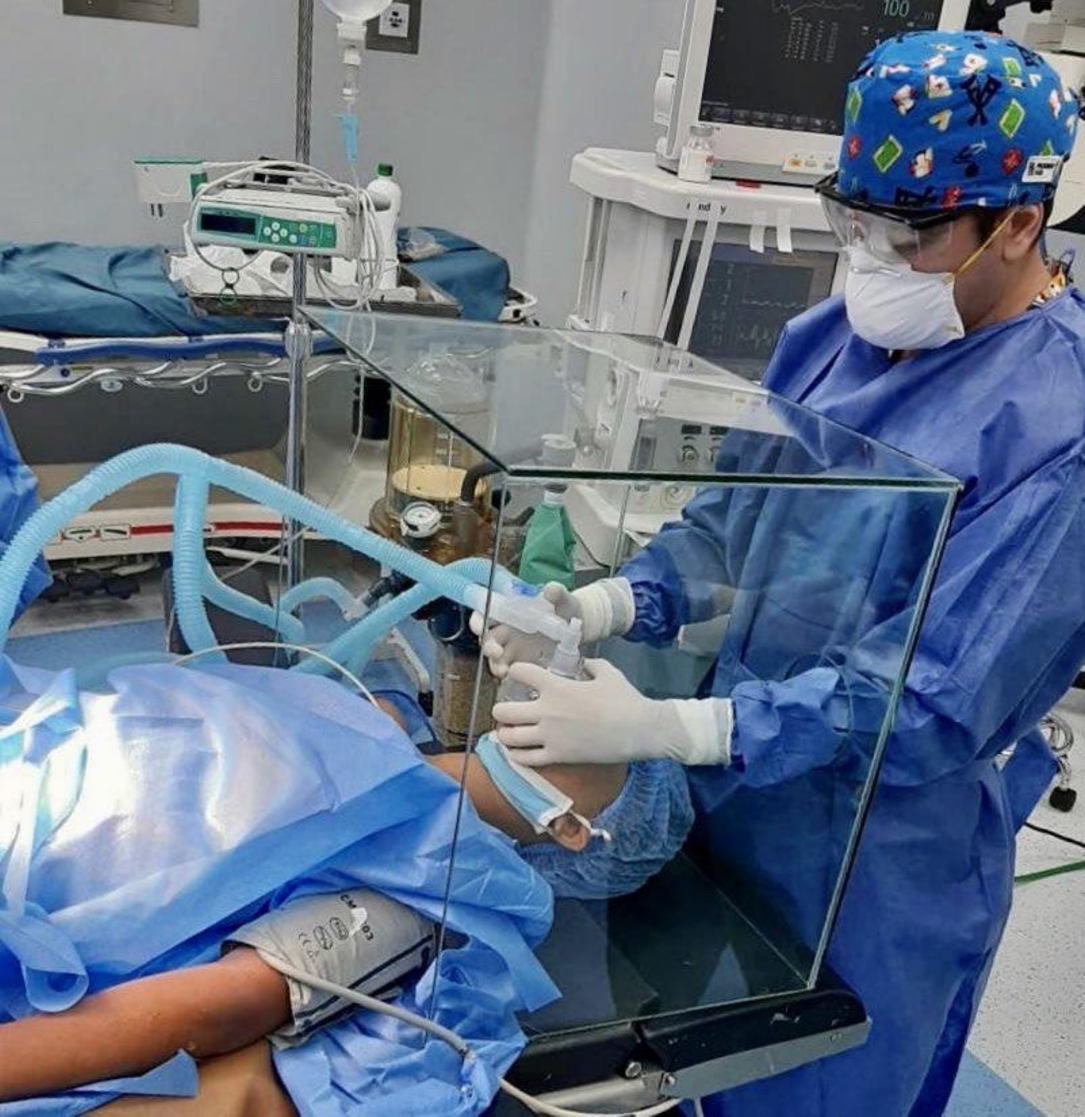
Aspects of Endoscopic Surgery.

Consequently, we describe the most up-to-date information, data, and recommendations on protection of personnel in the operating theater, and how to minimize the risk of subsequent contagion, as follows:

1. Preparation before surgery [16, 17]
  - Preparation of patients with suspected and/or confirmed COVID-19
    □ Validly informed consent, which includes the risk of exposure to COVID and potential consequences.
    □ Surgically treat only high-priority and emergency cases during the COVID pandemic.
    □ Consider elderly patients with comorbidity with severe risk of COVID infection and a fatal outcome.
  - Preparation of the operating theater [16, 17]
    □ Allow **minimum personnel** in the ward, even during the intubation procedure.
    □ Use a **smoke** extractor when using electrocauterization.
    □ Consider **avoiding laparoscopy**.
  - Preparation of the surgical team
    □ **Personal protection equipment** for each procedure performed on a COVID-19 positive patient or with suspicion of COVID-19.
    □ Respirators/N95 masks.
    □ Disposable masks or respirators.
    □ Additional resources on PPE.
    □ The fit test is essential to ensure that masks fit properly and minimize exposure.
    □ Contagion with the COVID-19 virus by aerosolization and Pilger droplets are significant risks for surgical personnel.
    □ Surgeons and personnel not necessary for intubation should remain outside the operating theater until the process of anesthesia and intubation is completed for patients with conformed or suspicion of COVID-19.
    □ Bear in mind how long COVID-19 may remain infectious on different surfaces (for example, cardboard 1 day, plastic 3-4 days).
2. Intraoperative management [16, 17]
  - Anesthetic management
    □ The type of anesthesia should be chosen based on the patient's conditions.
    □ The risk of contagion by aerosolization is increased with procedures like endotracheal intubation and tracheostomy and during pneumoperitoneum evacuation and aspiration of bodily fluids during laparoscopic surgery.
    □ Negative pressure should be maintained in operating theaters.
    □ Avoid changes of operating theater personnel (Figures 2A and B).
  - Aspects of laparoscopic and robot-assisted surgery
    □ Laparoscopic surgery may be associated with a greater quantity of smoke particles.
    □ Surgical smoke is released at low pressure in several stages of surgery.
    □ Do not insert 8 mm instruments in a 12 mm da Vinci trocar without a reduction.
    □ Do not insert a 5 mm instrument in a 12 mm da Vinci trocar even with the reduction in place.
    □ The lowest intraabdominal pressure permitted is recommended with the use of integrated intelligent insufflation systems.
  - Aspects of endoscopic surgery
    □ All procedures should be considered high risk.
    □ There is a link between urine leakage and virus transmission. However, although evidence of transmission of the disease via urine has not yet been confirmed, urine sampling (for urine culture, bars, and other analyses), urethral catheterization, and endoscopic procedures should be performed with caution.
    □ Irrigating fluid evacuated during endourological procedures should be collected through a closed system.
    □ Surfaces must be cleaned rapidly using suitable absorbents and decontaminated with chlorine (5000-10000 mg / L) or another suitable disinfectant (Figure-3).
3. Postoperative management [16, 17]
  - If it proves necessary to move a patient positive for COVID-19 or with suspected infection to a recovery area or ICU after surgery, the minimum possible number of personnel should participate in the move.
  - Personnel should use PPE and should not use the same equipment used in the surgery
  - Close the laminar flow and air supply in the operating theater
  - Sanitize the operating theater with peroxyacetic acid and reuse after 2 hours.

### Looking forward

Standard guidelines and procedures should be established to detect infectious diseases at an early stage, to opportunely announce pathogens, pathways of transmission, diagnosis, and treatment among healthcare professionals. Also, improvement in professional practice as an essential part of continuing medical education in all medical and public healthcare institutions is another critical step to reduce the rate of infection among healthcare personnel. Doctors, regardless of their areas of practice, should conduct routine emergency drills for infectious diseases, receive periodic professional training in protection against occupational risks. Especially, medical personnel involved in management of infectious diseases must be well trained in proper use of PPE, and the certificate of continuing education may be mandatory for key healthcare personnel or personnel in all medical institutions. Also, ease of access to mental health services for HP should be assured throughout their professional career, especially during the crisis when they need relief from anxiety and stress.

With stabilization of epidemics and measures taken by decision makers, the scarcity of PPE in China was attenuated significantly in mid-February [18]. However, the COVID-19 outbreak alerts us that a carefully planned stockpile of PPE and other essential supplies is key in effective preparation for infectious diseases and for the healthcare team to function optimally [19]. Because an epidemic may affect a broad population, availability and proper use of PPE, such as N95 respirators, facemasks, surgical gowns, and gloves, are crucial to protect the health of HP [20]. While it is very hard to predict a widespread epidemic outbreak, all healthcare centers should stockpile a certain amount of critical PPE to guarantee an adequate supply from the outset. Furthermore, it is also important to establish a centralized and coordinated supply network for emergency PPE between central and local governments, medical care facilities, and medical teams, to meet demand for consumable and durable supplies in a prolonged generalized epidemic.

## CONCLUSIONS

As urologists, we need to adapt our clinical practice to the new outlook facing us under the COVID-19 pandemic, anticipate that, as social distancing measures are lifted and until a vaccine is available, the risk of contagion will remain high for the majority of healthcare personnel, and therefore we must conduct our routine clinical and surgical urological activities without losing sight of that risk and taking appropriate and validated measures to minimize the risk of contagion among medical personnel.
